# Salivary DNA Methylation as an Epigenetic Biomarker for Head and Neck Cancer. Part I: A Diagnostic Accuracy Meta-Analysis

**DOI:** 10.3390/jpm11060568

**Published:** 2021-06-17

**Authors:** Óscar Rapado-González, Cristina Martínez-Reglero, Ángel Salgado-Barreira, Laura Muinelo-Romay, Juan Muinelo-Lorenzo, Rafael López-López, Ángel Díaz-Lagares, María Mercedes Suárez-Cunqueiro

**Affiliations:** 1 Department of Surgery and Medical-Surgical Specialties, Medicine and Dentistry School, Universidade de Santiago de Compostela, 15782 Santiago de Compostela, Spain; oscar.rapado@rai.usc.es (Ó.R.-G.); juanmuinelo@hotmail.com (J.M.-L.); 2 Translational Medical Oncology Group (Oncomet), Liquid Biopsy Analysis Unit, Health Research Institute of Santiago (IDIS), 15706 Santiago de Compostela, Spain; lmuirom@gmail.com; 3 Centro de Investigación Biomédica en Red de Cáncer (CIBERONC), Instituto de Salud Carlos III, 28029 Madrid, Spain; rafael.lopez.lopez@sergas.es (R.L.-L.); angel.diaz.lagares@sergas.es (Á.D.-L.); 4 Methodology and Statistics Unit, Galicia Sur Health Research Institute (IISGS), 36312 Vigo, Spain; cristina.martinez@iisgaliciasur.es (C.M.-R.); angel.salgado.barreira@sergas.es (Á.S.-B.); 5 Translational Medical Oncology Group (Oncomet), Health Research Institute of Santiago (IDIS), Complexo Hospitalario Universitario de Santiago de Compostela (SERGAS), 15706 Santiago de Compostela, Spain; 6 Cancer Epigenomics, Translational Medical Oncology Group (Oncomet), Health Research Institute of Santiago (IDIS), University Clinical Hospital of Santiago (CHUS/SERGAS), 15706 Santiago de Compostela, Spain

**Keywords:** DNA methylation, epigenetics, head and neck cancer, saliva, biomarkers, liquid biopsy, meta-analysis

## Abstract

DNA hypermethylation is an important epigenetic mechanism for gene expression inactivation in head and neck cancer (HNC). Saliva has emerged as a novel liquid biopsy representing a potential source of biomarkers. We performed a comprehensive meta-analysis to evaluate the overall diagnostic accuracy of salivary DNA methylation for detecting HNC. PubMed EMBASE, Web of Science, LILACS, and the Cochrane Library were searched. Study quality was assessed by the Quality Assessment for Studies of Diagnostic Accuracy-2, and sensitivity, specificity, positive likelihood ratio (PLR), negative likelihood ratio (NLR), diagnostic odds ratio (dOR), and their corresponding 95% confidence intervals (CIs) were calculated using a bivariate random-effect meta-analysis model. Meta-regression and subgroup analyses were performed to assess heterogeneity. Eighty-four study units from 18 articles with 8368 subjects were included. The pooled sensitivity and specificity of salivary DNA methylation were 0.39 and 0.87, respectively, while PLR and NLR were 3.68 and 0.63, respectively. The overall area under the curve (AUC) was 0.81 and the dOR was 8.34. The combination of methylated genes showed higher diagnostic accuracy (AUC, 0.92 and dOR, 36.97) than individual gene analysis (AUC, 0.77 and dOR, 6.02). These findings provide evidence regarding the potential clinical application of salivary DNA methylation for HNC diagnosis.

## 1. Introduction

Head and neck cancer (HNC) comprises a heterogenous group of epithelial malignancies arising from mucosal linings of the oral cavity, oropharynx, larynx, and hypopharynx. According to data from the World Health Organization’s GLOBOCAN network, HNC is highly prevalent worldwide, accounting for an estimated 890,000 new cases and 450,000 deaths in 2018 [1]. Despite improvements in diagnosis and therapeutic strategies, the 5-year survival rate for HNC has remained around 50% for the last decade. Unfortunately, HNC patients are frequently diagnosed in advanced stages involving a high risk of locoregional recurrence and distant metastasis [2,3]. Therefore, it is necessary to identify new biomarkers for diagnosis and prognosis to allow early detection and improved overall survival. HNC arises through multistep carcinogenic pathways as a result of cumulative genetic and epigenetic aberrations resulting from risk factors including alcohol and tobacco consumption, human papillomavirus infection, chronic inflammation, and genetic predisposition, and leading to reduced tumor suppressor gene function as well as oncogene activation [2,4]. In recent years, accumulating scientific evidence has highlighted the important role in tumorigenesis of epigenetic mechanisms, which represent a cancer hallmark [5,6]. Epigenetic alterations such as DNA methylation, histone covalent modifications, chromatin remodeling, and non-coding RNAs have been implicated in the landscape of phenotypical changes occurring in a wide variety of malignancies, including HNC [7]. DNA hypermethylation has been shown to be an important epigenetic mechanism for gene expression inactivation. The hypermethylation of cytosine–phosphodiester bond–guanine (CpG) islands within promoter regions plays an important role in carcinogenesis through the transcriptional silencing of different tumor suppressor genes or dysfunction in DNA repair genes [7,8]. Several studies have focused on the identification of aberrant promoter methylation patterns in HNC tissue and liquid biopsies [9,10]. Moreover, a number of investigations have detected promoter hypermethylation in various genes using saliva from HNC patients [10,11,12]. Therefore, salivary DNA hypermethylation represents a promising biomarker for non-invasively diagnosing HNC.

DNA methylation is a heritable and stable epigenetic mechanism implicated in the regulation of gene expression that plays an important role during normal development, regulating X chromosome inactivation, genomic imprinting, and preventing the transcription of DNA repetitive sequences, inserted viral sequences, and transposons [8,13]. DNA methylation is characterized by the covalent addition of a methyl group to the 5’-position of the pyrimidine ring of cytosines by DNA methyltransferases, giving rise to 5-methylcytosine. This enzymatic process occurs predominantly within CpG dinucleotides which are concentrated at CpG-rich DNA stretches named CpG islands (CGIs), which overlap the promoter region of 60–70% of protein-coding genes [14]. In the human genome, approximately 80% of CpG dinucleotides are heavily methylated whereas CGIs in gene promoters are mostly unmethylated, allowing active gene transcription [13]. Dysregulation of DNA methylation has been found to be involved in several diseases, representing an early epigenetic event in carcinogenesis. These alterations of normal DNA methylation patterns in cancer have been characterized as global hypomethylation and gene-specific hypermethylation. The global hypomethylation of repetitive sequences and transposable elements within the genome induces genomic instability and mutagenesis. In this line, the loss of DNA methylation may also activate latent viral sequences, promoting carcinogenesis. By contrast, in addition to global hypomethylation, aberrant promoter hypermethylation can drive the inactivation of key tumor suppressor genes, which are unmethylated in non-malignant tissues [15]. In this sense, although silencing by DNA hypermethylation of some genes is common in many types of tumors, the methylation profile of gene promoters is different for each human cancer, allowing the identification of cancer-specific hypermethylation patterns [16]. Although tissue biopsy of the primary tumor or metastatic lesions remains the gold standard method for diagnosis, DNA methylation biomarkers can be assessed in different liquid biopsies, representing a non-invasive alternative for early cancer detection [10,17,18].

Recently, saliva has emerged as an attractive liquid biopsy for genomic and epigenomic analysis. Saliva-based liquid biopsy is a fast, reliable, cost-effective, and non-invasive approach to analyze epigenetic alterations involved in the onset and course of the disease. Some researchers have found comparable methylation profiles between saliva and tissue, representing a non-invasive alternative for epigenomic profiling [19,20]. Additionally, although similar methylation DNA patterns have been reported between saliva and blood [21,22], methylation differences between both biofluids can be identified due to tumor shedding and tissue-specific methylation [23,24]. Focusing on promoter hypermethylation, a number of investigations have detected various methylated genes in saliva, representing a promising biomarker for non-invasive HNC detection [10,11,12].

The purpose of this systematic review and meta-analysis was to summarize the results of published clinical studies to assess the overall diagnostic accuracy of salivary DNA hypermethylation for discriminating HNC.

## 2. Materials and Methods

### 2.1. Protocol and Registration

This study was conducted according to Preferred Reporting Items for Systematic Reviews and Meta-analysis (PRISMA) guidelines [25], and the protocol was registered with the International Prospective Register of Systematic Reviews (reference No. CRD42020199114).

### 2.2. Search Strategy and Study Selection

The systematic literature search of eligible articles published up to 27 August 2020 was carried out without language restrictions using PubMed, EMBASE, Web of Science, LILACS, and the Cochrane Library. The search strategy was based on the following combinations of keywords and medical subject headings: (methylation OR hypermethylation OR epigenomics) AND (saliva OR oral rinse OR mouthwash) AND (head and neck cancer OR head and neck neoplasm OR head and neck carcinoma OR head and neck squamous cell carcinoma OR HNSCC OR oral cancer OR pharyngeal cancer OR laryngeal cancer). Studies were screened based on title and abstract, and eligible manuscripts were retrieved for full-text review. In addition, reference lists from each original and review article were searched manually in order to find further relevant studies. The literature search was performed independently by two investigators (ORG and MMSC), and disagreements during the selection process were resolved by consensus. The studies selected by means of the search strategy and other references were managed using RefWorks software (https://www.refworks.com/content/path_learn/faqs.asp, accessed 28 October 2020), and duplicate items were removed using the associated tools.

### 2.3. Selection Criteria

The inclusion criteria were as follows: (1) studies that evaluated the diagnostic accuracy of gene promoter hypermethylation in saliva samples from HNC patients; (2) inclusion of a control group consisting of healthy controls; (3) sufficient data for generating a two-by-two (2 × 2) contingency table containing true positive (TP), false positive (FP), true negative (TN), and false negative (FN) values. The exclusion criteria were as follows: (1) reviews, letters, personal opinions, book chapters, case reports, conference abstracts, and meetings; (2) duplicate publications; (3) in vitro and in vivo animal experiments.

### 2.4. Data Extraction

All eligible studies were assessed independently by two investigators (ORG and MMSC) and data were extracted using a pre-established form designed on a Microsoft Excel spreadsheet (Microsoft Corp. Redmond, WA, USA). Any disagreement among reviewers was resolved by consensus. The following information was extracted from each study: name of first author, year of publication, country, anatomic tumor location, number of cases and controls, positive methylated cases, positive methylated controls, method for DNA methylation detection, type of saliva sample (saliva or oral rinse), methylated gene names, and statistical analysis outcomes, including diagnostic accuracy and cut-off values. If the required data were incomplete, attempts were made to contact the authors to obtain the missing information. We defined “study unit” as the analysis of a relationship between gene promoter hypermethylation and HNC. Therefore, a single publication could potentially include more than one study unit as a result of reporting promoter hypermethylation for multiple genes.

### 2.5. Quality Assessment of Individual Studies

Following Healthcare Research and Quality Agency recommendations, two independent researchers (ADL and LMR) applied the Quality Assessment of Diagnostic Accuracy Studies-2 checklist (QUADAS-2) [26]. Any discrepancies were resolved by a third reviewer (MMSC). The QUADAS-2 checklist assesses study quality by analyzing four key domains: (1) patient selection, (2) index tests, (3) reference tests, and (4) flow and times. Risk of bias and applicability concerns for each domain were assessed as “low”, “high”, or “unclear”. One point was assigned to each item assessed as “low”. Thus, articles were grouped into the following quality categories based on their cumulative score: “high” quality (6–7 points), “moderate” quality (4–5 points), and “low” quality (0–3 points).

### 2.6. Statistical Analysis

MetaDiSc software (v.1.4) [27], free R software (v.3.4.4; https://www.r-project.org, accessed 30 November 2020), and STATA (v.14.0; https://www.stata.com, accessed 30 November 2020) were used to carry out statistical analysis. The numbers of TP, FP, FN, and TN in each study unit in the diagnostic meta-analysis were extracted to calculate pooled sensitivity [TP/(TP + FN)], specificity [TN/(TN + FP)], positive likelihood ratio (PLR) [(sensitivity/(1 − sensitivity)], negative likelihood ratio (NLR), [(1 − specificity)/specificity)], diagnostic odds ratio (dOR), and their corresponding 95% confidence intervals (CIs) using a bivariate random or fixed effect meta-analysis model. The pooled diagnostic performance of salivary DNA promoter hypermethylation for HNC detection was determined by plotting the summary receiver operator characteristic (SROC) curve and calculating the area under the SROC curve (AUC). Heterogeneity analysis was used to identify factors influencing accuracy indicators and the statistical model applied [27]. Spearman’s correlation analysis and ROC plane plots were used to assess heterogeneity due to the threshold effect. Cochran’s Q statistic test-based chi-squared test and I^2^ statistics were used to assess non-threshold heterogeneity. When I^2^ > 50% and/or *p* < 0.05 for the Cochran’s Q test, heterogeneity was considered to be significant. The DerSimonian and Laird random effects model was applied when heterogeneity was significant; otherwise, we applied the Mantel–Haenszel fixed effects model. Potential sources of non-threshold heterogeneity were explored by meta-regression and subgroup analyses. In addition, the predictive value of post-salivary DNA promoter hypermethylation for HNC diagnosis was evaluated by Fagan’s nomogram. Post-test probability was calculated using Bayes theorem under the assumption of prior probabilities of 25%, 50%, and 75%, respectively [28]. Deeks’ funnel plot asymmetry test [29] was used to ascertain publication bias (statistical significance: *p* < 0.05).

## 3. Results

### 3.1. Study Selection

**Figure 1:**
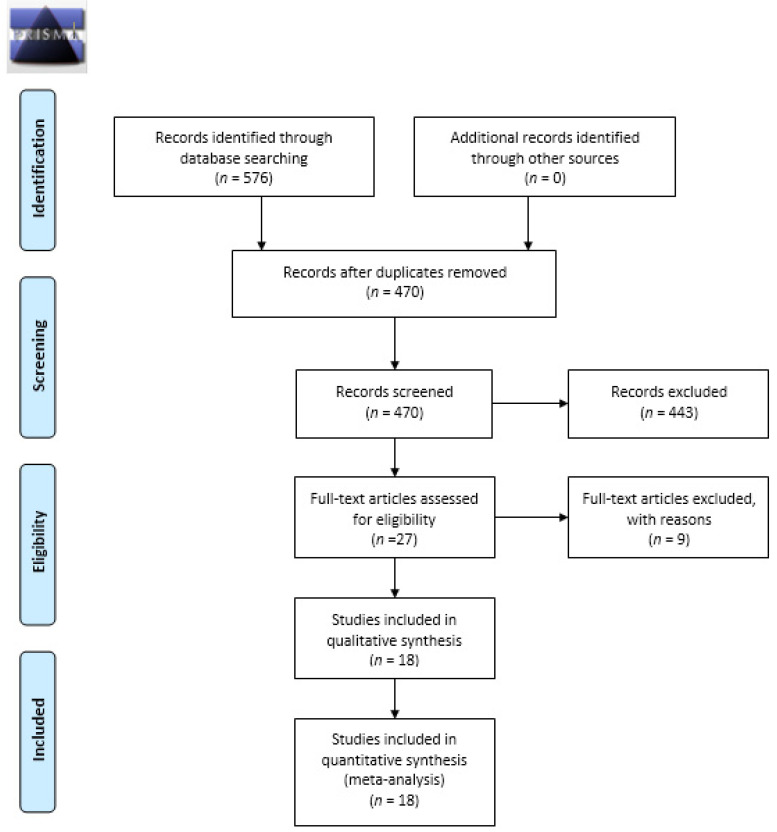
PRISMA flow diagram.

A PRISMA flowchart for the literature identification and selection process is shown in Figure 1. A total of 576 studies were identified based on the search strategy across the five electronic databases, which was reduced to 470 after removing duplicates. After title and abstract review, 27 articles were submitted for full-text reading, of which nine were excluded for the following reasons: non-independent cancer group (two articles); reviews, letters, personal opinions, book chapters, case reports, conference abstracts, and meetings (three articles); absence of a healthy control group (one article); saliva enriched with brush oral cytology (two articles); and insufficient information for meta-analysis (one article). In the end, 18 articles met the inclusion criteria for final analysis [10,11,12,30,31,32,33,34,35,36,37,38,39,40,41,42,43,44]. 

### 3.2. Characteristics of Included Studies

The characteristics of the included studies are shown in Table 1. The 18 articles comprised a total of 84 study units, including 4758 HNC patients and 3605 healthy individuals (the sample size ranged from 13 [31] to 210 [41]). All articles were published between 2001 and 2020, and studies were conducted in the following geographical regions: the United States (5), Australia (3), Thailand (2), Japan (2), France (1), Brazil (1), India (1), Taiwan (1), Colombia (1), and Italy (1). Saliva samples included oral rinses (12) and whole saliva (6). Salivary DNA methylation was detected by different methods, including methylation-specific polymerase chain reaction (MSP) (11) and quantitative MSP (7). A total of 34 different genes were identified in the studies. Five studies evaluated the methylation status of a single gene [31,34,36,39,41] and 13 studies evaluated two or more genes [10,11,12,30,32,33,35,37,38,40,42,43,44]. Ten studies focused on gene promoter methylation panels combining two to four genes, whereas eight studies evaluated only single genes.

### 3.3. Quality Assessment of the Included Studies

All included articles were evaluated for risk of bias using the QUADAS-2 checklist (Figure S1). The major risk of bias in this study was patient selection domain, as 13 out of 18 publications were unclear or lacked detail on whether the patient sample was consecutive or random. Additionally, 14 out of 18 studies did not provide a detailed description of the inclusion/exclusion criteria. Moreover, there was high risk of bias in the index test, since some studies lacked a setting threshold. All domains were considered to have a low risk of bias in terms of applicability concern. All studies were of moderate-to-high quality, with an average QUADAS-2 score of 5.6.

### 3.4. Diagnostic Accuracy of Salivary DNA Promoter Hypermethylation

**Figure 2:**
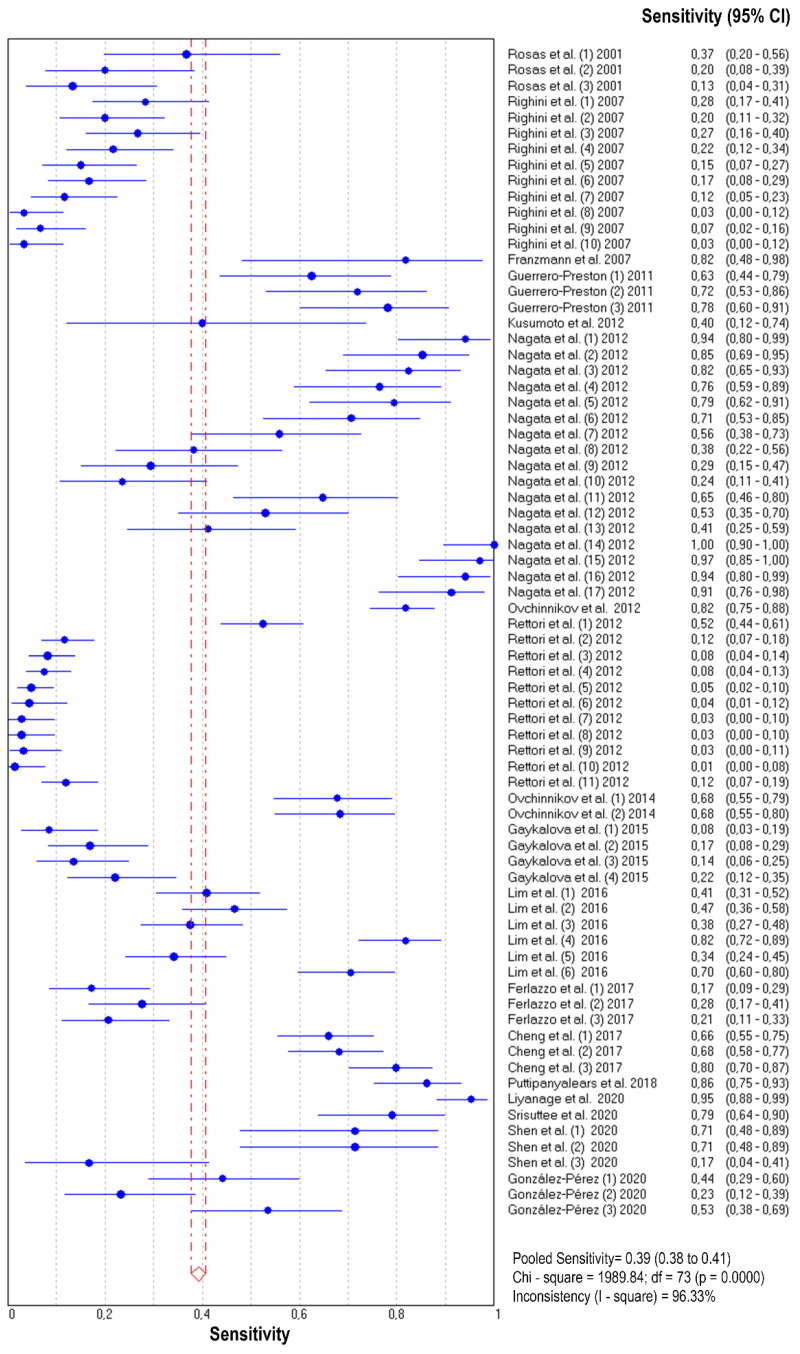
Forest plot of sensitivities from test accuracy studies of salivary DNA methylation for predicting HNC diagnosis.

**Figure 3:**
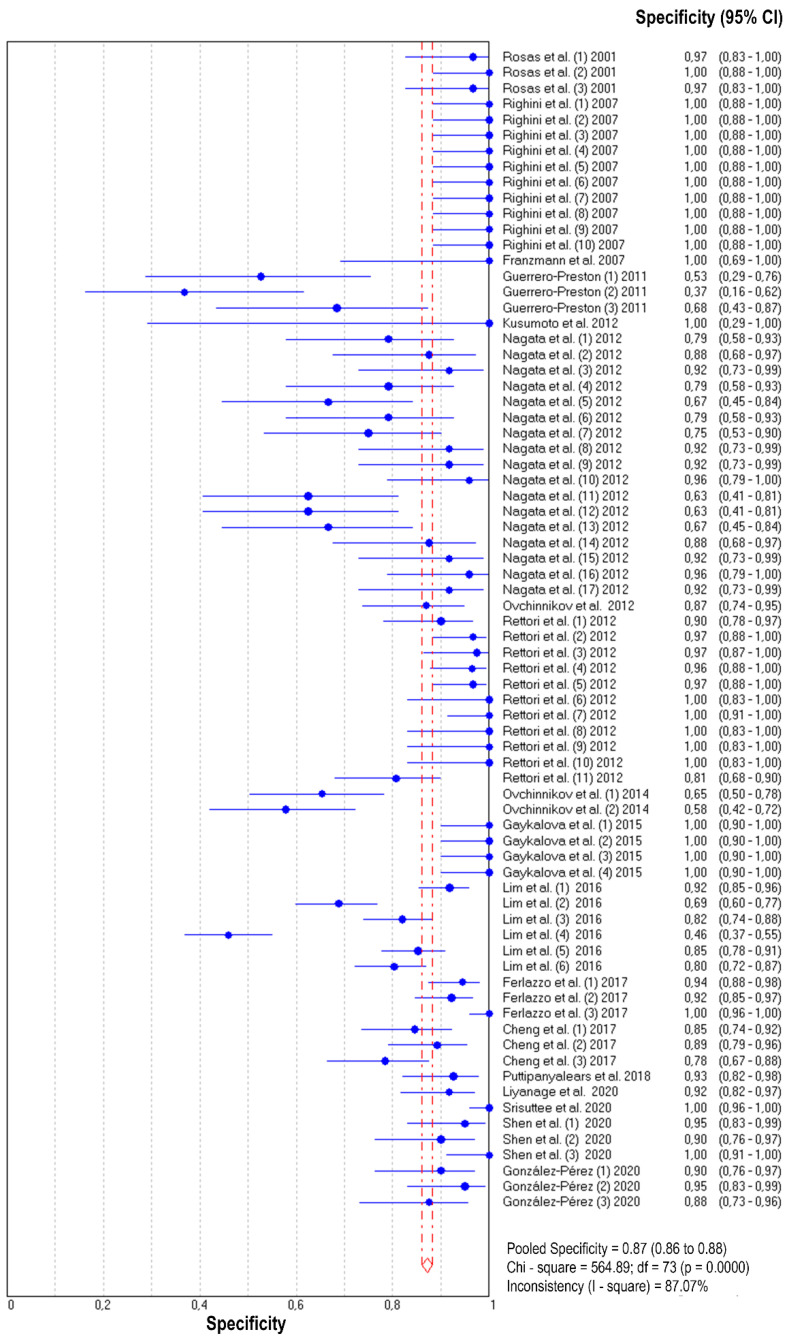
Forest plot of specificities from test accuracy studies of salivary DNA methylation for predicting HNC diagnosis.

**Figure 4:**
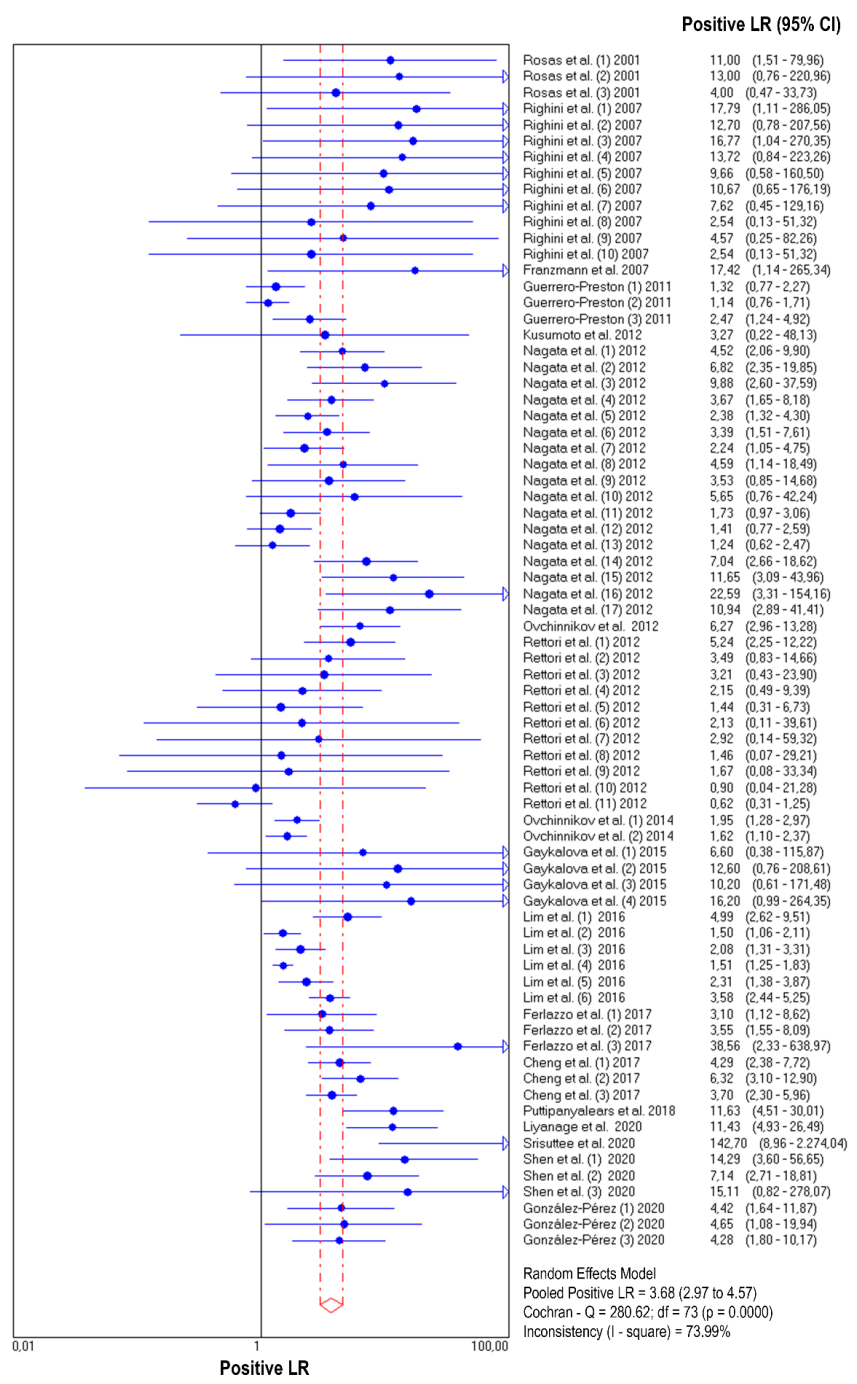
Forest plot of likelihood ratios for positive test results from salivary DNA methylation studies for predicting HNC diagnosis.

**Figure 5:**
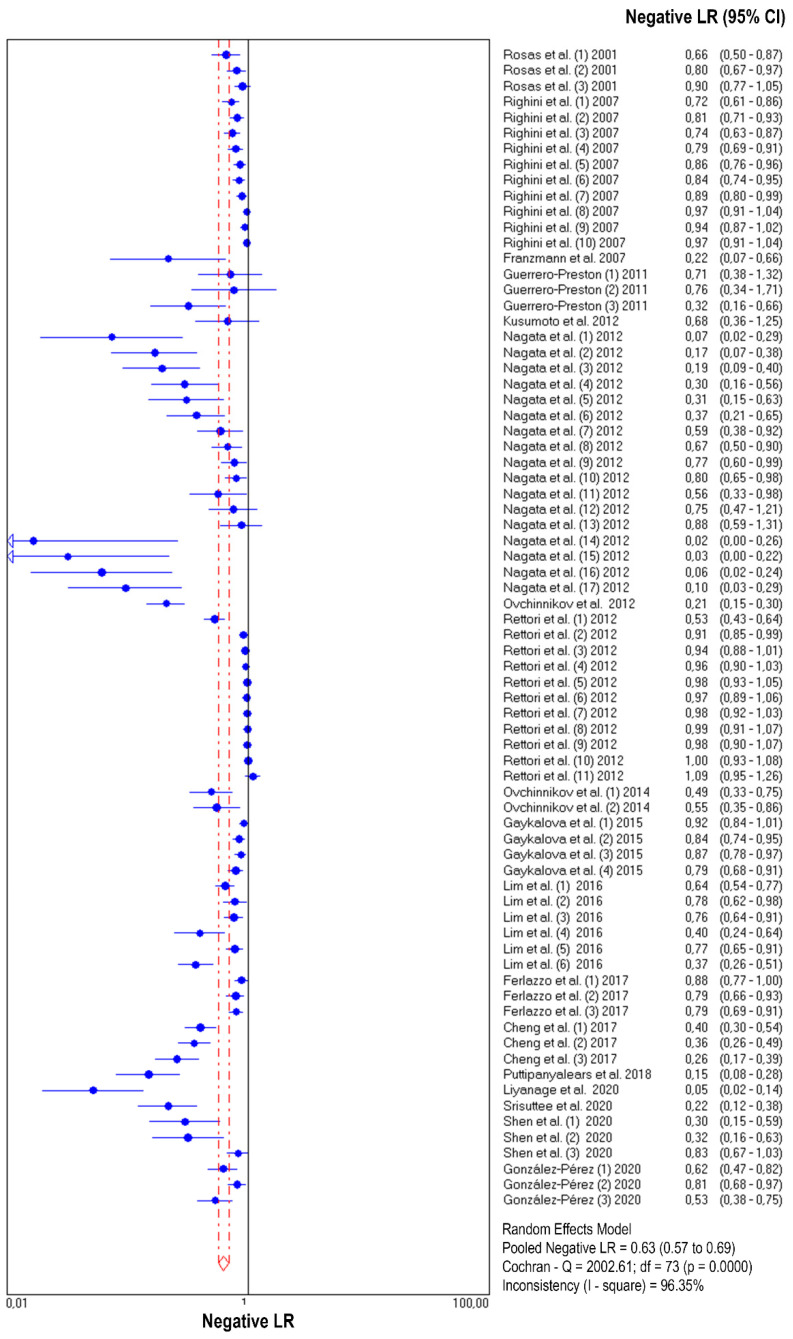
Forest plot of likelihood ratios for negative test results from salivary DNA methylation studies for predicting HNC diagnosis.

**Figure 6:**
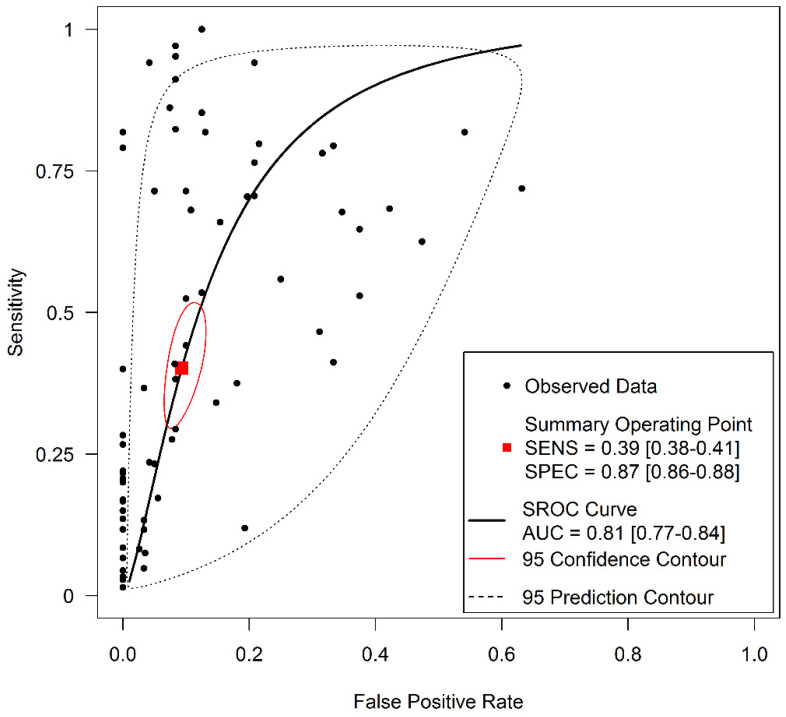
SROC curve with pooled estimates of sensitivity, specificity, and the AUC for all included studies of the salivary DNA methylation studies for detecting HNC.

The diagnostic accuracy (sensitivity, specificity, and 95% confidence interval) of each study unit (*n* = 74) included in this meta-analysis is shown in Figure 2 and Figure 3. The pooled sensitivity and specificity of salivary DNA hypermethylation genes in the diagnosis of HNC were 0.39 (95% CI: 0.38–0.41) and 0.87 (95% CI: 0.86–0.88), respectively. The PLR and NLR were 3.68 (95% CI: 2.97–4.57) and 0.63 (95% CI: 0.57–0.69) (Figure 4 and Figure 5), respectively; the summary dOR was 8.34 (95% CI: 6.10–11.39) (Figure S2); and the area under the SROC was 0.81 (95% CI 0.77–0.84) (Figure 6). As shown in Fagan’s nomogram (Figure S3), given a pre-test probability of 27.8%, a positive measurement leads to a post-test cancer probability of 59%, whereas a negative measurement leads to a post-test probability of 20%.

### 3.5. Heterogeneity and Subgroup Analysis

As shown in Figure 2, Figure 3, Figure 4 and Figure 5 and Figure S2, significant heterogeneity was observed regarding the pooled sensitivity (I^2^ = 96.33%; *p* < 0.001), specificity (I^2^ = 87.07%; *p* < 0.001), PLR (I^2^ = 73.99%; *p* < 0.001), NLR (I^2^ = 96.35%; *p* < 0.001), and dOR (I^2^ = 71.83%; *p* < 0.001). The representation of accuracy estimates from each study in the SROC space revealed a typical pattern of a “shoulder arm”, suggesting the presence of a threshold effect (Figure S4). Moreover, Spearman’s correlation coefficient between the logit of the true positive rate and the logit of the false positive rate was 0.633 (*p* = 0.000), which showed further indication of a threshold effect. In addition to the variations due to the threshold effect, meta-regression was performed to determine the possible sources of heterogeneity using the following covariates as predictor variables: sample type, sample size, anatomic tumor location, DNA methylation methods, and methylation gene profiling. The results indicated that anatomic tumor location (*p* = 0.002) and gene profiling (*p* < 0.001) were potential sources of heterogeneity in this study (Table S1). Consequently, subgroup analysis based on anatomic tumor location (HNC vs. oral cancer vs. oropharyngeal cancer) and gene profiling (single vs. combination of genes) was performed. As shown in Table S2, the results indicated similar accuracy for salivary methylated genes in oral cancer (sensitivity, 0.63; specificity, 0.87; PLR, 4.02; NLR, 0.40; dOR, 13.07; AUC, 0.88) and oropharyngeal cancer (sensitivity, 0.70; specificity, 0.86; PLR, 3.67; NLR, 0.41; dOR, 13.26; AUC, 0.87). However, differences in diagnostic accuracy were observed in the HNC group (sensitivity, 0.31; specificity, 0.86; PLR, 3.03; NLR, 0.75; dOR, 5.78; AUC, 0.81). When basing the meta-analysis on gene profile, the combination of methylated genes showed higher diagnostic accuracy (sensitivity, 0.73; specificity, 0.88; PLR, 5.76; NLR, 0.22; dOR, 36.97; AUC, 0.92) compared to individual genes (sensitivity, 0.32; specificity, 0.87; PLR, 3.17; NLR, 0.71; dOR, 6.02; AUC, 0.77). Although the meta-regression results were negative for other covariates, we conducted subgroup analyses based on these factors to further explore the diagnostic potential of salivary DNA methylated genes (Table S2).

### 3.6. Publication Bias

The potential publication bias in each salivary DNA methylation study was explored by Deeks’ funnel plot asymmetry test, which yielded a slope coefficient *p*-value of 0.711 overall. This indication of a symmetric data pattern suggests the absence of publication bias (Figure S5).

## 4. Discussion

Over the last few years, saliva has aroused great interest in the scientific community due to its potential as a non-invasive liquid biopsy in cancer. Several studies have evidenced the diagnostic capability of salivary biomarkers for diagnosing both HNC [45] and tumors distant from the oral cavity [46]. In this sense, a wide variety of biomolecules have been assessed as tumor biomarkers using saliva-omics approaches, including genomic, epigenomic, transcriptomic, metabolomic, proteomic, and microbiomic technologies [47]. In the field of epigenomics, DNA promoter hypermethylation represents one of the most intensively studied epigenetic alterations in human cancer. Promoter hypermethylation of critical pathway genes has been recognized as an important epigenetic mechanism of carcinogenesis [13]. Its potential role as an early diagnostic biomarker stems from the fact that gene promoter hypermethylation is an early event in cancer development [13]. DNA hypermethylation as a common event in cancer plays an important role in HNC development and progression [7]. The detection of DNA methylation in body fluids has emerged as an opportunity to assess the methylation status non-invasively and cost-effectively. In this line, several studies have investigated the promoter methylation of different tumor-suppressor genes in saliva from HNC patients [10,11,12,38]. Therefore, salivary DNA methylation biomarkers could be potentially used in the screening and early detection of HNC.

To the best of our knowledge, this study represents the first meta-analysis evaluating the diagnostic accuracy of promoter hypermethylation genes in saliva for differentiating HNC patients from healthy individuals. The present analysis included a total of 18 articles (84 study units) involving 4758 HNC patients and 3605 healthy individuals. According to QUADAS-2 quality evaluation, most of the included studies were of moderate quality. As for the overall accuracy of salivary hypermethylated genes for discriminating HNC from healthy individuals, the pooled diagnostic parameters of sensitivity, specificity, and AUC values were 0.39, 0.87, and 0.81, respectively. The summary dOR of 8.34 reflects the diagnostic capacity of salivary hypermethylated genes for HNC. The pooled PLR value of 3.68 indicates that a person testing positive had approximately 3.68 times higher probability of having cancer than a healthy individual. On the other hand, the pooled NLR indicated that a person testing negative had a 63% probability of not having cancer. Additionally, given a pre-test probability of 27.8% in Fagan’s nomogram, correct HNC diagnosis increased to 59% after a positive test, and reduced to 20% after a negative test. Overall, these results show a low sensitivity for HNC detection, indicating a high false negative rate. Therefore, the salivary gene methylation evaluated in this meta-analysis presented limitations as a screening biomarker for HNC. However, the diagnostic specificity of gene methylation for HNC was very high, suggesting that detection in saliva may aid assessment in HNC diagnosis. New molecular biology techniques such as next-generation sequencing platforms and digital PCR represent an opportunity for improving the sensitivity of methylation assays and for discovering new methylation patterns.

Due to the fact that heterogeneity is inherent to any diagnostic accuracy meta-analysis, an evaluation of the reasons contributing to inconsistencies across studies should be carried out. In the present research, overall heterogeneity among studies was high, so a bivariate random effects model was applied. This significant heterogeneity was reflected numerically in Cochran’s Q test and the I^2^ statistic. Further exploration of the heterogeneity revealed the presence of a threshold effect. The threshold effect is a major source of heterogeneity in meta-analyses of diagnostic tests. It arises from differences in sensitivities and specificities or likelihood ratios due to different cut-offs among studies for defining positive (or negative) test results [27]. In the present meta-analysis, the threshold effect was suggested by the visual inspection of accuracy estimates in forest plots where increasing specificities with decreasing sensitivities were observed. Later, the ROC plane and the Spearman correlation test also indicated the presence of the threshold effect. The variations in accuracy estimates among different studies could be due to a number of reasons other than the threshold, such as study population (anatomic tumor location, TNM staging), index test (differences in technology, assays), reference standard, and study design. Therefore, heterogeneity should be explored by relating study level co-variates to an accuracy measure by meta-regression techniques [27]. In the present study, meta-regression was performed to test the effect of sample type, sample size, anatomic tumor location, DNA methylation method, and methylated gene profiling. The results point to anatomic tumor location and gene profiling strategy as possible causes of heterogeneity. Stratified analysis by anatomic tumor location showed that salivary gene methylation had a higher diagnostic accuracy for discriminating oral (AUC = 0.88) and oropharyngeal (AUC = 0.87) tumors than overall HNC (AUC = 0.81). The explanation for these findings may be that tumors located in the oral cavity and oropharynx release more tumor cells directly into saliva. With respect to gene profiling strategy, subgroup analysis showed that single genes presented low sensitivity, but were highly specific to cancer tissue. The combination of salivary methylated genes had better diagnostic accuracy than single gene-based tests, with a dOR of 36.97 vs. 6.02 and AUC of 0.92 vs. 0.77, respectively, demonstrating that the use of salivary methylated gene panels as biomarkers may increase HNC detection accuracy without decreasing specificity. We also conducted subgroup analyses based on sample type, sample size, and DNA methylation method but no significant differences in diagnostic accuracy were observed. Future studies should be conducted to clarify the impact of these factors on the diagnostic potential of salivary DNA methylation.

The current meta-analysis is not free of limitations. Firstly, this meta-analysis included case–control studies, but none was multicenter. Moreover, no randomized controlled trials exist on this topic. Secondly, considerable heterogeneity was observed among the included studies. Although we examined the sources of heterogeneity in five variables, we were not able to explore other demographic and clinicopathological factors due to lack of information. Thirdly, studies involving saliva samples only evaluated some of the genes methylated in HNC tissue. The remaining genes should also be tested in saliva to determine their diagnostic potential. Lastly, confounding variables such as gender, age, lifestyle (tobacco and alcohol), and diet were not considered in most of the included studies. Due to these limitations, future research based on large-scale prospective diagnostic studies involving multiple health centers would contribute to further evaluating the clinical utility of salivary gene promoter methylation for HNC diagnosis. Furthermore, better comparison among future studies would benefit from standardization of analytic strategies and cut-off selection.

## 5. Conclusions

Our meta-analysis suggests that the detection of DNA promoter hypermethylation in saliva is a promising biomarker for HNC diagnosis, mainly in oral and oropharyngeal tumors. The use of salivary hypermethylated gene panels improves diagnostic accuracy with respect to single-gene analysis. This meta-analysis could provide valuable insights into methodology design for further research studies.

## Figures and Tables

**Table 1:** Summary of descriptive characteristics of included studies.

First Author	Anatomic Tumor Location	Type of Sample	Method	Biomarker	Cancer Group N (M+)	Control Group N (M+)
Rosas 2001	HNC	Oral rinse (NaCl)	MSP	p16	30 (11+)	30 (1+)
				DAPK	30 (6+)	30 (0+)
				MGMT	30 (4+)	30 (1+)
Righini 2007	HNC	Oral rinse (NaCl)	MSP	TIMP3	60 (17+)	30 (0+)
				ECAD	60 (12+)	
				p16	60 (16+)	
				MGMT	60 (13+)	
				DAPK	60 (9+)	
				RASSF1A	60 (10+)	
				p15	60 (7+)	
				p14	60 (2+)	
				APC	60 (4+)	
				FHIT	60 (2+)	
				hMLH1	60 (0+)	
Franzmann 2007	HNC	Oral rinse (NaCl)	MSP	CD44	11 (9+)	10 (0+)
Guerrero-Preston 2011	HNC	Oral rinse (NaCl)	qMSP	HOXA9	32 (20+)	19 (9+)
				NID2	32 (23+)	19 (12+)
				HOXA9+NID2	32 (25+)	19 (6+)
	OC			HOXA9	16 (11+)	19 (9+)
				NID2	16 (14+)	19 (12+)
				HOXA9+NID2	16 (14+)	19 (6+)
	OPC			HOXA9	16 (9+)	19 (9+)
				NID2	16 (9+)	19 (12+)
				HOXA9+NID2	16 (11+)	19 (6+)
Nagata 2011	OC	Oral rinse (NaCl)	MSP	ECAD	34 (32+)	24 (5+)
				TMEFF2	34 (29+)	24 (3+)
				RARβ	34 (28+)	24 (2+)
				MGMT	34 (26+)	24 (5+)
				FHIT	34 (27+)	24 (8+)
				WIF1	34 (24+)	24 (5+)
				DAPK	34 (19+)	24 (6+)
				p16	34 (13+)	24 (2+)
				HIN	34 (10+)	24 (2+)
				TIMP3	34 (8+)	24 (1+)
				p15	34 (22+)	24 (9+)
				APC	34 (18+)	24 (9+)
				SPARC	34 (14+)	24 (8+)
				ECAD+TMEFF2+RARβ+MGMT	34 (34+)	24 (3+)
				ECAD+TMEFF2+MGMT	34 (33+)	24 (2+)
				ECAD+TMEFF2+RARβ	34 (32+)	24 (1+)
				ECAD+RARβ+MGMT	34 (31+)	24 (2+)
Ovchinnikov 2012	OC	Saliva	Nested MSP	p16+RASSF1A+DAPK1	143 (117+)	46 (6+)
Rettori 2012	HNC	Oral rinse (NaCl)	qMSP	DCC	143 (75+)	50 (5+)
				CCNA1	146 (17+)	60 (2+)
				DAPK	146 (12+)	39 (1+)
				MGMT	146 (11+)	57 (2+)
				TIMP3	146 (7+)	60 (2+)
				MINT31	68 (3+)	20 (0+)
				AIM1	71 (2+)	41 (0+)
				SFRP1	71 (2+)	20 (0+)
				APC	62 (2+)	20 (0+)
				CDKN2A	69 (1+)	20 (0+)
				HIN1	134 (16+)	57 (11+)
				CCNA1+DAPK+DCC+MGMT+TIMP3	NA	NA
				CCNA1+DAPK+MGMT+TIMP3	NA	NA
				CCNA1+DAPK+MGMT	NA	NA
				CCNA1+MGMT+TIMP3	NA	NA
				CCNA1+DAPK+TIMP3	NA	NA
				DAPK+MGMT+TIMP3	NA	NA
				CCNA1+MGMT	NA	NA
				CCNA1+DAPK	NA	NA
				CCNA1+TIMP3	NA	NA
Ksumoto 2012	OC	Oral rinse	MSP	p16	10 (4+)	3 (0+)
Ovchinnikov 2014	HNC	Saliva	MSP	PCQAP5’	62 (42+)	49 (17+)
				PCQAP3’	60 (41+)	45 (19+)
Gaykalova 2015	HNC	Oral rinse	qMSP	ZNF14	59 (5+)	35 (0+)
				ZNF160	59 (10+)	35 (0+)
				ZNF420	59 (8+)	35 (0+)
				ZNF14+ZNF160+ZNF420	59 (13+)	35 (0+)
Lim 2016	HNC	Saliva	MSP	RASSF1α	88 (36+)	122 (10+)
				p16	88 (41+)	122 (38+)
				TIMP3	88 (33+)	122 (22+)
				PCQAP5’	88 (72+)	122 (66+)
				PCQAP3’	88 (30+)	122 (18+)
				RASSF1α+p16+TIMP3+PCQAP5’+PCQAP3’	88 (62+)	122 (24+)
Ferlazzo 2017	OC	Saliva (Oragene DNA kit)	MSP	P16	58 (10+)	90 (5+)
				MGMT	58 (16+)	90 (7+)
				P16 + MGMT	58 (12+)	90 (0+)
Cheng 2017	OC	Oral rinse (0.12% clorhexidine)	qMSP	ZNF582	94 (62+)	65 (10+)
				PAX1	94 (64+)	65 (7+)
				ZNF582+PAX1	94 (75+)	65 (14+)
Puttipanyalears 2018	HNC	Oral rinse (NaCl)	qMSP	TRH	66 (57+)	54 (4+)
	OC				42 (37+)	
	OPC				24 (20+)	
Liyanage 2020	HNC	Saliva	MSP	p16	88 (62+)	NA
				RASSF1 α	88 (59+)	NA
				TIMP3	88 (68+)	NA
				PCQAP/MED15	88 (66+)	NA
				p16+RASSF1α+TIMP3+PCQAP	84 (80+)	60 (5+)
	OC			p16	54 (39+)	NA
				RASSF1α	54 (37+)	NA
				TIMP3	54 (43+)	NA
				PCQAP/MED15	54 (43+)	NA
				p16+RASSF1α+TIMP3+PCQAP	54 (46+)	60 (5+)
	OPC			p16	34 (23+)	NA
				RASSF1α	34 (22+)	NA
				TIMP3	34 (25+)	NA
				PCQAP/MED15	34 (23+)	NA
				p16+RASSF1α+TIMP3+PCQAP	34 (34+)	60 (5+)
Srisuttee 2020	OC	Oral rinse (NaCl)	qMSP	NID2	43 (34+)	90 (0+)
Shen 2020	OPC	Oral rinse (NaCl)	qMSP	EDNRB	21 (15+)	40 (2+)
				PAX5	21 (15+)	40 (4+)
				p16	21 (3+)	40 (0+)
González-Pérez 2020	OC	Saliva	MSP	p16	43 (19+)	40 (4+)
				RASSF1A	43 (10+)	40 (2+)
				p16+RASSF1A	43 (23+)	40 (5+)

*Abbreviations:* HNC = head and neck cancer; OC = oral cancer; OPC = oropharyngeal cancer; MSP = methylation-specific polymerase chain reaction; qMSP = quantitative MSP; NA = not available.

## Supplementary Materials

The following are available online at https://www.mdpi.com/article/10.3390/jpm11060568/s1, Figure S1: Quality assessment of the included studies according to Quality Assessment of Diagnostic Accuracy Studies-2 (QUADAS-2) criteria, Figure S2: Forest plot of pooled dOR salivary DNA methylation for the diagnosis of HNC, Figure S3: Fagan’s monogram evaluating the clinical utility of salivary DNA methylation for differentiating HNC patients, Figure S4: Representation of sensitivity against (1-specificity) in ROC space for each study of salivary methylation in the diagnosis of HNC, Figure S5: Deeks’ funnel plot asymmetry test for the assessment of potential bias of included studies, Table S1: Results of meta-regression analysis, Table S2: Subgroup analysis of salivary DNA methylation for HNC detection based on different covariates.
