# Supplementary material for: Salivary DNA Methylation as an Epigenetic Biomarker for Head and Neck Cancer. Part I: A Diagnostic Accuracy Meta-Analysis
Source: J Pers Med. 2021 Jun 17;11(6):568. doi: 10.3390/jpm11060568 (PMC8233749; doi:10.3390/jpm11060568)
Supplement: Supplementary file 1 [file jpm-11-00568-s001.zip › jpm-1227608-supplementary.pdf]

Journal of *Personalized Medicine*

MDPI

Supplementary Material

Salivary DNA methylation as an epigenetic biomarker for head and neck cancer. Part I: A diagnostic accuracy meta-analysis

Óscar Rapado-González, Cristina Martínez-Reglero, Ángel Salgado-Barreira, Laura Muinelo-Romay, Juan Muinelo-Lorenzo, Rafael López-López, Ángel Díaz-Lagares and María Mercedes Suárez-Cunqueiro

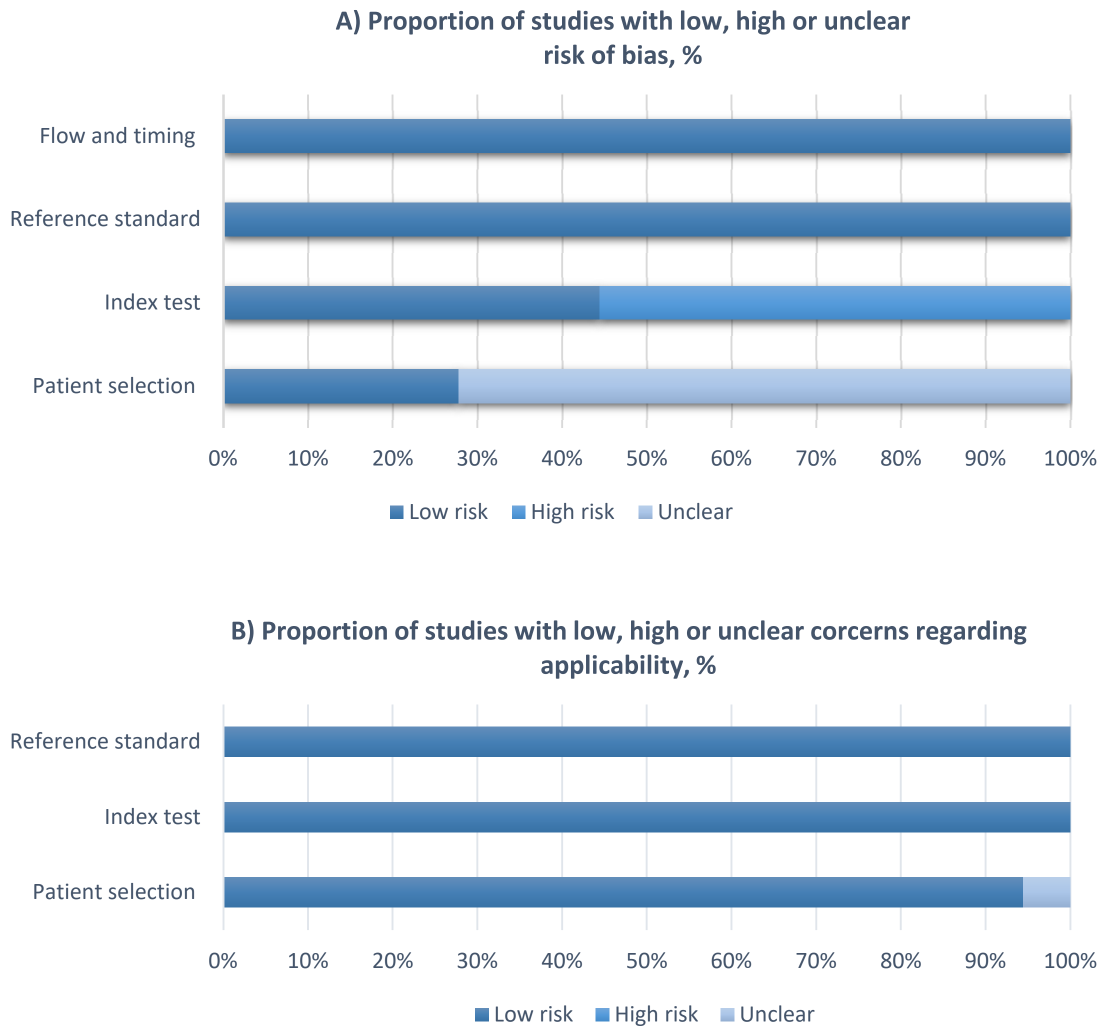

Figure S1. Quality assessment of the included studies according to Quality Assessment of Diagnostic Accuracy Studies-2 (QUADAS-2) criteria. (**A**) Proportion of studies with low, high, or unclear risk of bias. (**B**) Proportion of studies with low, high, or unclear concerns regarding applicability.

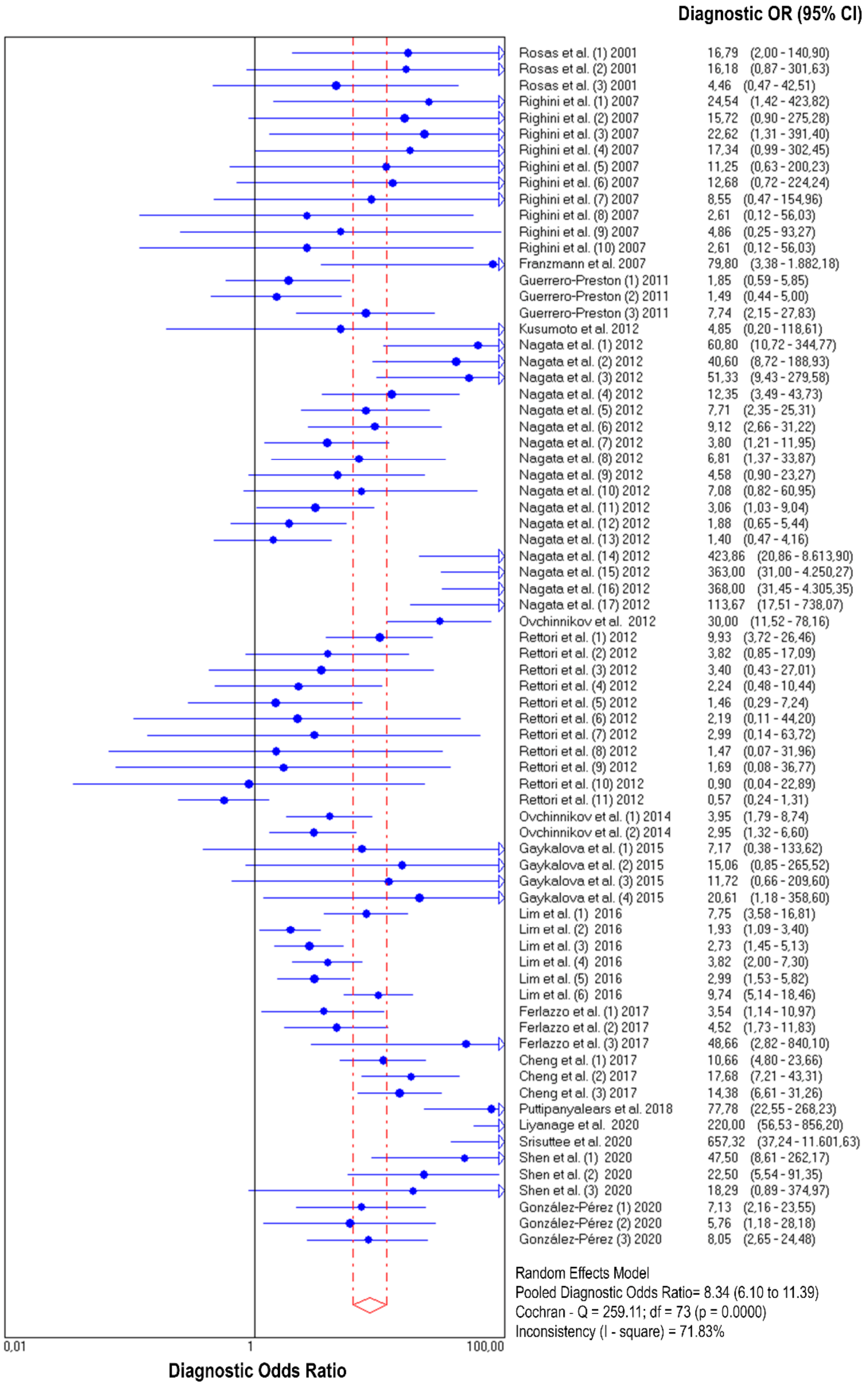

Figure S2. Forest plot of pooled dOR salivary DNA methylation for the diagnosis of HNC. *Abbreviations*: dOR = diagnostic Odds Ratio; HNC = head and neck cancer.

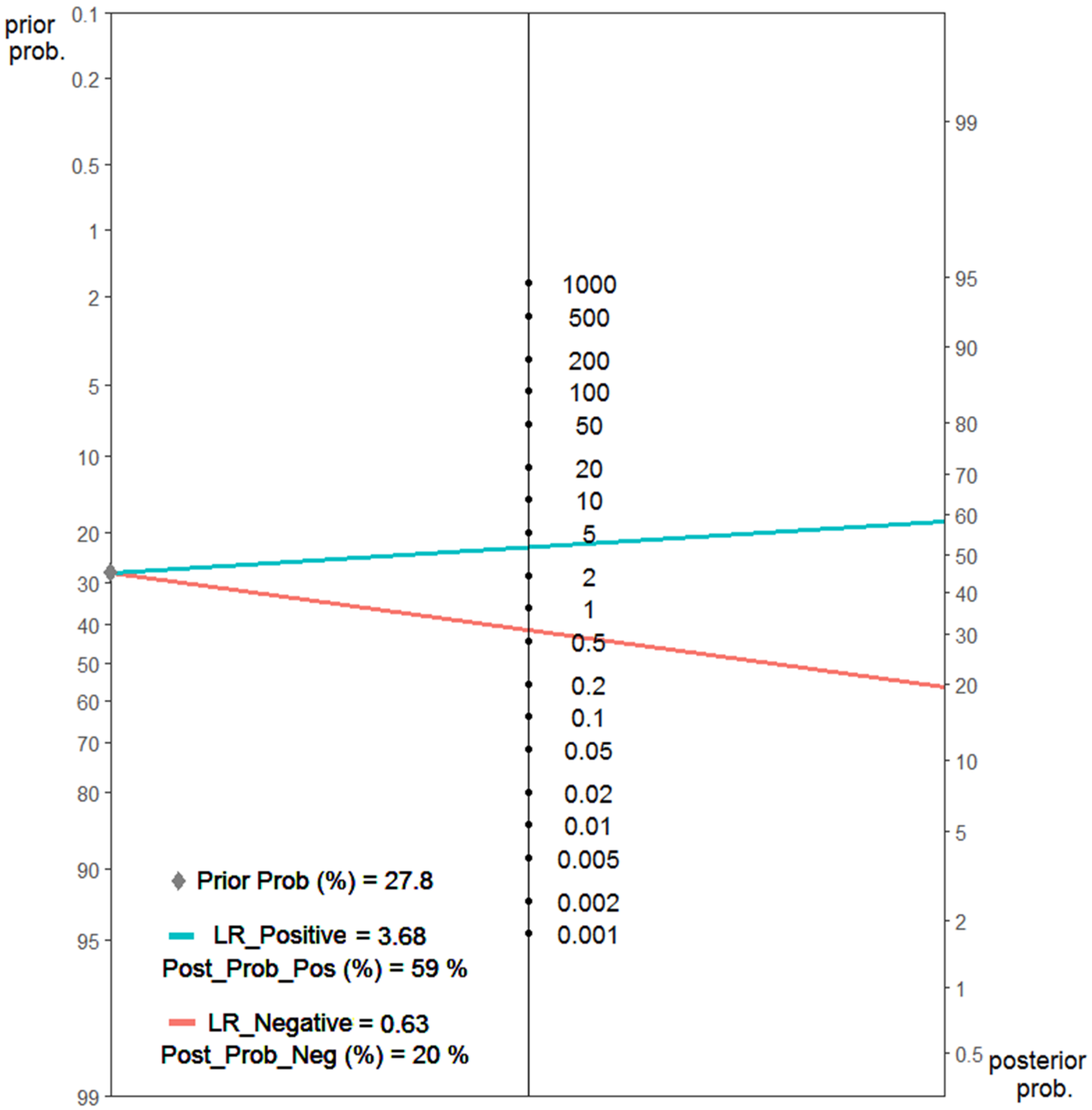

Figure S3. Fagan´s monogram evaluating the clinical utility of salivary DNA methylation for differentiating HNC patients. *Abbreviations*: HNC = head and neck cancer.

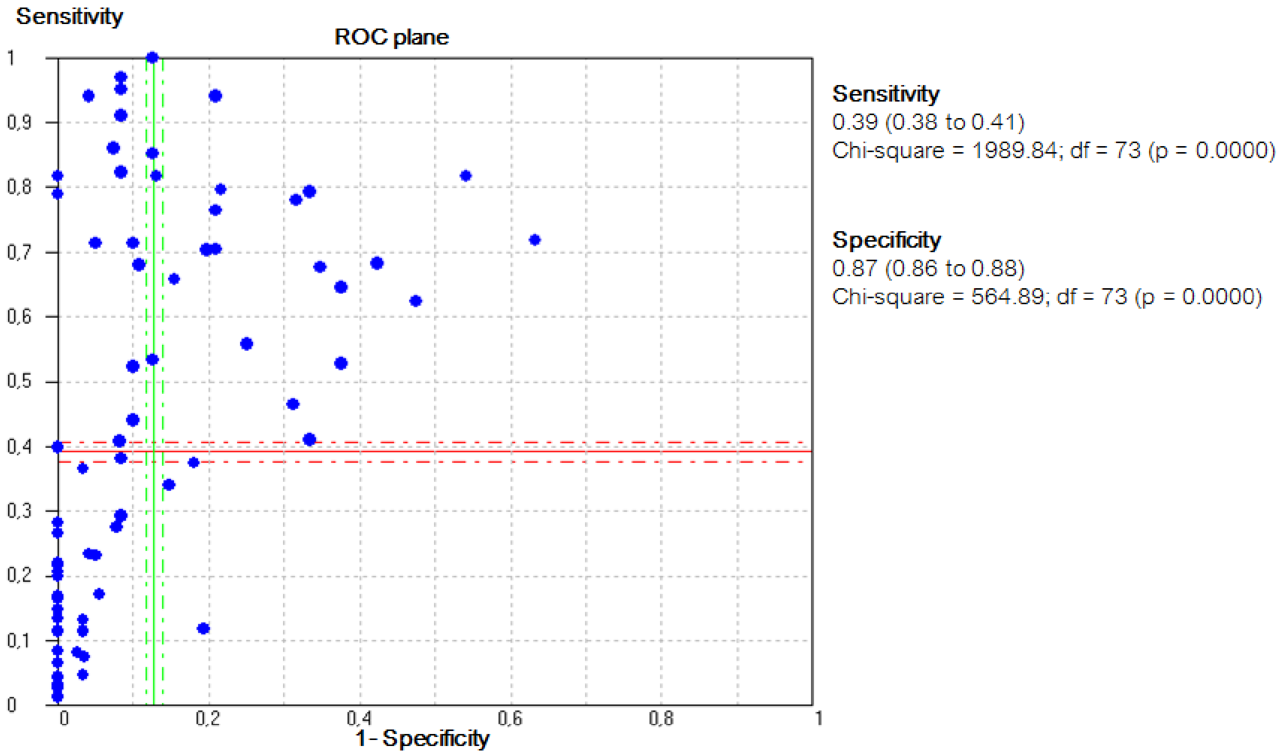

Figure S4. Representation of sensitivity against (1-specificity) in ROC space for each study of salivary methylation in the diagnosis of HNC. *Abbreviations*: ROC = summary receiver operator characteristic; HNC = head and neck cancer.

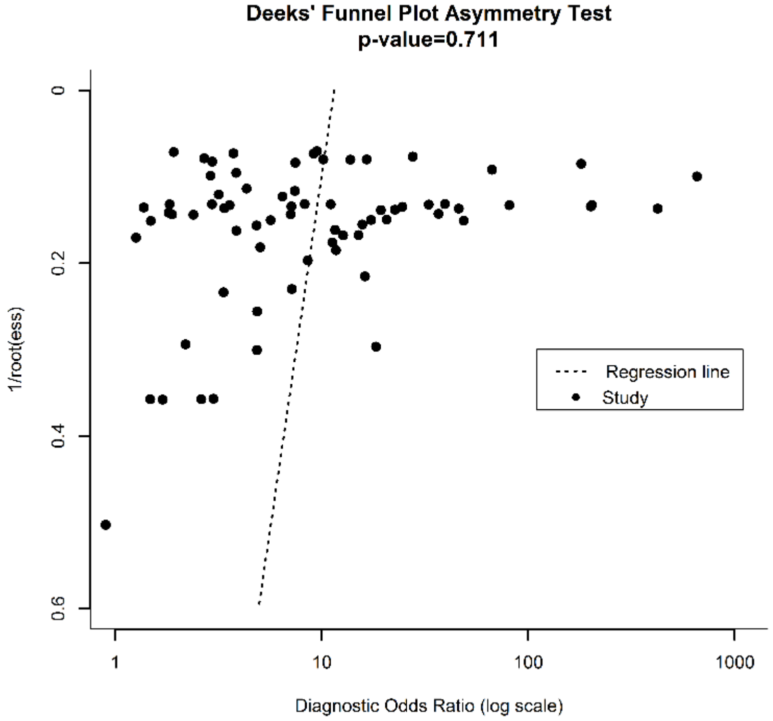

Figure S5. Deeks' funnel plot asymmetry test for the assessment of potential bias of included studies.

Table S1. Results of regression meta-analysis.

Var	Coeff.	*p*-value	RDOR	[95%Cl]
Cte.	-1.549	0.0592	-	-
S	0.023	0.7139	-	-
Sample type	0.739	0.1276	2.09	(0.81;5.44)
Anatomic tumor location	0.631	0.0204	1.88	(1.11;3.19)
Technique	-0.483	0.2752	0.62	(0.26;1.48)
Gene profiling	1.502	0.0000	4.49	(2.34;8.62)
Sample size	0.371	0.3493	1.45	(0.66;3.18)

Var	Coeff.	*p*-value	RDOR	[95%Cl]
Cte.	-0.864	0.1082	-	-
S	0.021	0.7316	-	-
Anatomic tumor location	0.731	0.0016	2.08	(1.33;3.24)
Gene profiling	1.421	0.0000	4.14	(2.19;7.84)

No. studies = 74.
Filter OFF.
Add ½ to all cells of the studies with zero.
Abbreviations: CI = confidence interval; Coeff. = coefficient; Std. Err. = standard error; RDOR = relative diagnostic odds ratios.

Table S2. Subgroup analysis of salivary DNA methylation for HNC detection based on different covariates.

Subgroups	No of study units	Sensitivity (95% CI)	I^2(%)	Specificity (95% CI)	I^2 (%)	PLR (95% CI)	I^2 (%)	NLR (95% CI)	I^2 (%)	DOR (95% CI)	I^2 (%)	AUC (95% CI)
Total	74	0.39 (0.38-0.41)	96.33	0.87 (0.86-0.88)	87.07	3.68 (2.97-4.57)	73.99	0.63 (0.57-0.69)	96.35	8.34 (6.10-11.38)	71.83	0.81 (0.77-0.84)
Sample type												
Saliva	16	0.55 (0.52-0.57)	94.98	0.81 (0.79-0.83)	92.30	3.04 (2.20-4.21)	82.52	0.57 (0.47-0.69)	90.55	6.33 (3.90-10.27)	79.34	0.78 (0.72-0.83)
Oral Rinse	58	0.34 (0.32-0.35)	96.28	0.91 (0.90-0.92)	81.02	4.07 (3.08-5.39)	66.18	0.65 (0.59-0.73)	96.39	9.42 (6.30-14.08)	67.84	0.82 (0.78-0.86)
Samples size												
>100	25	0.43 (0.41-0.45)	97.64	0.84 (0.83-0.86)	91.61	3.24 (2.35-4.46)	84.32	0.56 (0.46-0.67)	97.79	6.88 (4.24-11.19)	84.15	0.79 (0.73-0.84)
<100	49	0.35 (0.33-0.40)	94.89	0.91 (0.94-0.93)	80.63	4.12 (3.06-5.54)	60.01	0.68 (0.61-0.76)	94.46	9.58 (6.44-14.27)	53.11	0.82 (0.78-0.86)
Anatomic Tumor Location												
HNC	43	0.31 (0.29-0.33)	96.77	0.86 (0.85-0.87)	90.43	3.03 (2.27-4.03)	73.63	0.75 (0.96-0.82)	95.39	5.78 (3.86-8.67)	70.11	0.81 (0.75-0.88)
OC	33	0.63 (0.60-0.65)	92.22	0.87 (0.85-0.89)	76.63	4.02 (2.99-5.41)	73.79	0.40 (0.31-0.50)	92.45	13.07 (8.19-20.88)	73.08	0.88 (0.84-0.93)
OPC	8	0.70 (0.62-0.77)	86.26	0.86 (0.81-0.89)	88.41	3.67 (2.88-4.69)	88.67	0.41 (0.19-0.90)	90.75	13.26 (3.17-55.42)	83.12	0.87 (0.72-1.00)
Technique												
MSP	48	0.47 (0.45-0.49)	95.08	0.85 (0.83-0.86)	87.22	3.59 (2.81-4.57)	72.53	0.59 (0.52-0.67)	94.49	9.06 (6.30-13.03)	70.46	0.82 (0.78-0.85)
qMSP	26	0.29 (0.27-0.31)	97.15	0.92 (0.90-0.93)	84.79	3.65 (2.31-5.77)	76.18	0.69 (0.60-0.79)	97.12	6.81 (3.70-12.54)	74.82	0.78 (0.71-0.86)
Gene profiling												
Single gene	62	0.32 (0.31-0.34)	95.45	0.87 (0.86-0.88)	88.12	3.17 (2.53-3.97)	69.76	0.71 (0.66-0.77)	94.50	6.02 (4.45-8.13)	62.54	0.77 (0.73-0.81)
Combination gene	12	0.73 (0.69-0.76)	95.29	0.88 (0.58-0.91)	78.40	5.76 (3.92-8.48)	54.71	0.22 (0.12-0.40)	96.78	36.97 (16.81-81.32)	73.62	0.92 (0.88-0.96)

Abbreviations: AUC = area under the SROC; dOR = diagnostic Odds Ratio; PLR = positive likelihood ratio; NLR = negative likelihood ratio; CI = confidence interval; HNC = head and neck cancer; OC = oral cancer; OPC = oropharyngeal cancer; MSP = methylation-specific polymerase chain reaction; qMSP = quantitative-MSP.
